# Structural and functional characterization of IdiA/FutA (Tery_3377), an iron-binding protein from the ocean diazotroph *Trichodesmium erythraeum*

**DOI:** 10.1074/jbc.RA118.001929

**Published:** 2018-09-14

**Authors:** Despo Polyviou, Moritz M. Machelett, Andrew Hitchcock, Alison J. Baylay, Fraser MacMillan, C. Mark Moore, Thomas S. Bibby, Ivo Tews

**Affiliations:** From the ‡ Ocean and Earth Sciences, National Oceanography Centre Southampton, University of Southampton, Southampton SO14 3ZH, United Kingdom,; the § Department of Biological Sciences, Faculty of Natural and Environmental Sciences, Institute for Life Sciences, University of Southampton, Southampton SO17 1BJ, United Kingdom, and; the ¶ School of Chemistry, University of East Anglia, Norwich NR4 7TJ, United Kingdom

**Keywords:** ABC transporter, X-ray crystallography, iron, cyanobacteria, microscopy, diazotroph, iron deficiency

## Abstract

Atmospheric nitrogen fixation by photosynthetic cyanobacteria (diazotrophs) strongly influences oceanic primary production and in turn affects global biogeochemical cycles. Species of the genus *Trichodesmium* are major contributors to marine diazotrophy, accounting for a significant proportion of the fixed nitrogen in tropical and subtropical oceans. However, *Trichodesmium* spp. are metabolically constrained by the availability of iron, an essential element for both the photosynthetic apparatus and the nitrogenase enzyme. Survival strategies in low-iron environments are typically poorly characterized at the molecular level, because these bacteria are recalcitrant to genetic manipulation. Here, we studied a homolog of the iron deficiency-induced A (IdiA)/ferric uptake transporter A (FutA) protein, Tery_3377, which has been used as an *in situ* iron-stress biomarker. IdiA/FutA has an ambiguous function in cyanobacteria, with its homologs hypothesized to be involved in distinct processes depending on their cellular localization. Using signal sequence fusions to GFP and heterologous expression in the model cyanobacterium *Synechocystis* sp. PCC 6803, we show that Tery_3377 is targeted to the periplasm by the twin-arginine translocase and can complement the deletion of the native *Synechocystis* ferric-iron ABC transporter periplasmic binding protein (FutA2). EPR spectroscopy revealed that purified recombinant Tery_3377 has specificity for iron in the Fe^3+^ state, and an X-ray crystallography–determined structure uncovered a functional iron substrate–binding domain, with Fe^3+^ pentacoordinated by protein and buffer ligands. Our results support assignment of Tery_3377 as a functional FutA subunit of an Fe^3+^ ABC transporter but do not rule out dual IdiA function.

## Introduction

Photosynthetic organisms have a higher requirement for iron than heterotrophs because of the dependence of multiple components of the photosynthetic electron transport chain on this essential element (1, 2). This requirement is amplified in nitrogen-fixing cyanobacteria (diazotrophs) because iron is also required by the nitrogenase enzyme (3–5). Therefore, marine diazotrophic cyanobacteria species such as the globally significant *Trichodesmium* have developed diverse strategies that permit survival at the fluctuating or chronically depleted iron concentrations typically encountered in the surface waters of the tropical and subtropical oceans (5–9). *Trichodesmium* accounts for approximately half of the total marine nitrogen fixation (10), supplying both biologically available nitrogen and fixed carbon to oligotrophic ecosystems (11). Physiological experiments suggest *Trichodesmium* can acquire iron from diverse sources (7, 12), and analysis of its genome reveals homologs to iron-uptake systems, such as FeoAB for ferrous (Fe^2+^) iron uptake and the periplasmic ferric hydroxamate-binding protein FhuD (5). Although “omic” studies and similarity to homologous systems in other bacteria can be used to infer function, the inability to genetically engineer *Trichodesmium* means no iron-uptake mechanisms have been directly characterized *in vivo*, limiting our understanding of how this important species has adapted to acquire iron from its environment.

Ubiquitous in cyanobacterial genomes and present in *Trichodesmium* are homologs of a protein variably referred to in the literature as IdiA (iron deficiency-induced) or FutA (ferric uptake transporter). IdiA/FutA-like proteins are synthesized under iron limitation in marine and freshwater cyanobacteria (13, 14) and as such are commonly exploited as *in situ* markers of iron stress (15–17). Despite this, functional assignment of these proteins is presently ambiguous. Homologs can have an intracellular localization (18) and are proposed to be associated with the cytoplasmic side of the thylakoid membrane to protect the acceptor side of photosystem II (PSII)^4^ against oxidative damage during iron stress (18, 19). However, the same protein is also homologous to ferric binding proteins of ATP-binding cassette (ABC) transporters, found in some pathogenic bacteria and several cyanobacteria (20–22).

In the model freshwater cyanobacterium *Synechocystis* sp. PCC 6803 (hereafter *Synechocystis*), there are two characterized paralogs of IdiA/FutA, referred to as FutA1 (Slr1295) and FutA2 (Slr0513). FutA1 has a predominantly intracellular localization, associating with the thylakoid membrane, although it has also been found in the cytoplasmic membrane. In contrast, the FutA2 protein is a periplasmic substrate-binding protein associated with partner FutB and FutC subunits to form an active Fe^3+^ uptake ABC transporter (22–26).

*Trichodesmium erythraeum* IMS101 encodes only one IdiA/FutA homolog in its genome, Tery_3377 (17). Increased expression/production under iron stress has been reported at both the transcriptional (3, 17), and protein levels (5, 13). Tery_3377 is therefore thought to play an important role in iron homeostasis.

In this study we investigated the role of Tery_3377 to understand whether it is performing a periplasmic function (FutA2-like) or an intracellular function (FutA1-like). We produced Tery_3377 in *futA1* and *futA2* mutants in the genetically tractable model cyanobacterium *Synechocystis* sp. PCC 6803 testing for complementation of mutant phenotypes. We also used signal peptide fusions to GFP to give insight into the cellular localization of Tery_3377. Further, we overproduce and purify recombinant Tery_3377 and confirm ferric iron (Fe^3+^) coordination by electron paramagnetic resonance (EPR) spectroscopy and structure determination, classifying Tery_3377 as a class IV iron substrate–binding domain (27). This functional and structural investigation of Tery_3377 helps to characterize its role in the iron-stress physiology of a globally significant microbe and suggests a potential need to reassess the roles of FutA/IdiA family proteins more generally.

## Results

### Insights from sequence analysis

The protein sequence of the Tery_3377 protein is 58 and 57% identical to *Synechocystis* FutA1 and FutA2, respectively (Table S1). In *Synechocystis*, FutA1 and FutA2 are 52% identical to one another (21) yet appear to have distinct cellular function. Functional assignment based on primary sequence comparisons is therefore not possible. In addition, like the *Synechocystis futA1* and *futA2* genes, the Tery_3377 gene is located at an independent locus to genes encoding putative Fut ABC-transporter permease (FutB/Tery_3223) and ATPase subunits (FutC/Tery_3222), making functional inference from genomic context impossible.

**Figure 1.**
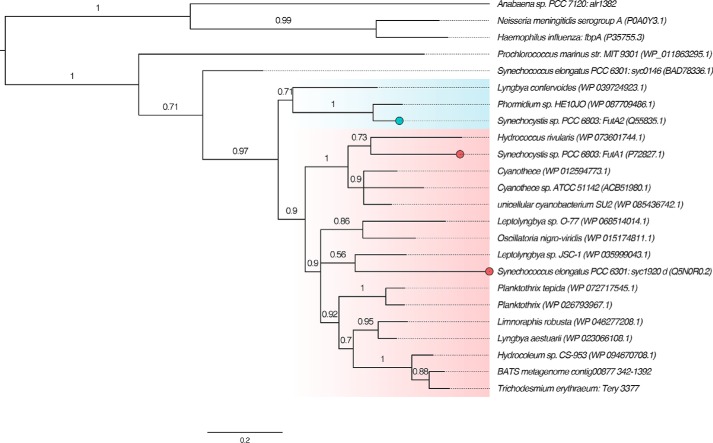
**Maximum likelihood phylogenetic analysis of Tery_3377 homologs, based on amino acid sequence.** Homologs of Tery_3377 and *Synechocystis* FutA1 and FutA2 were identified using BlastP (40) and aligned along with sequences from the cyanobacteria *S. elongatus* PCC 6301, *P. marinus* MIT 9301, and *Anabaena* PCC 7120, and the pathogenic bacteria *H. influenza* and *N. meningitides*. The graphical representation was produced using FigTree v1.4.3, and branch probabilities reported are calculated using an approximate likelihood ratio test (47). The *Synechocystis* FutA1 (*red*) and FutA2 (*blue*) proteins are separated into two groups, and Tery_3377 clusters within the FutA1-like group. *Filled circles* represent branches of the tree for which there is experimental evidence of intracellular (*red*) or iron transport (*blue*) function. Database accessions for each protein (SwissProt where possible, otherwise RefSeq) are given in *parentheses*. The *scale bar* represents the number of substitutions per site.

Phylogenetic analysis was carried out to investigate grouping of Tery_3377 with IdiA/FutA homologs (Fig. 1). A multiple sequence alignment was constructed from Tery_3377, FutA1, and FutA2, along with homologs from range of cyanobacterial species, and this was used to generate a maximum likelihood phylogenetic tree. The *Synechocystis* FutA1 and FutA2 proteins are separated into distinct branches within the tree (branch support 0.97, approximate likelihood ratio test). Tery_3377 appears to be more closely related to the intracellular FutA1 protein than to FutA2. To confirm placement of the Tery_3377 protein within the phylogeny, we searched for homologs in other publicly available *Trichodesmium* genomes/metagenomes. No Tery_3377 homolog could be detected in the draft genome of *Trichodesmium theibautii* H94 (Bioproject PRJNA278659); however, this genome is known to be incomplete (28). A homolog was found in a metagenomic dataset from the Bermuda Atlantic Time Series (Bioproject PRJNA336846). This protein (encoded between nucleotides 342 and 1392 on contig 00877) clustered with Tery_3377 (Fig. 1).

### The Tery_3377 signal peptide targets GFP to the periplasm

To investigate the cellular localization of Tery_3377, a synthetic gene encoding the predicted signal peptide (amino acids 1–31) and the first four residues of the mature protein (amino acids 32–35) fused to super-folder (sf)GFP was expressed in *Synechocystis* under the control of the *psbAII* promoter (Table S1 and Fig. S1). For comparison, the predicted *Synechocystis* FutA1 and FutA2 signal peptides were also fused to sfGFP and expressed from the *psbAII* promoter. The *Escherichia coli* TMAO reductase (TorA) signal peptide that has previously been shown to transport GFP/yellow fluorescent protein into the periplasm of *Synechocystis* was used as a positive control, and sfGFP alone, *i.e.* without signal sequence, acted as a marker of cytoplasmic expression (29, 30).

**Figure 2.**
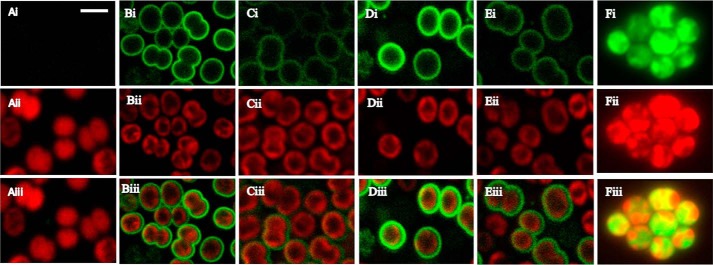
**Localization of signal sequence-GFP constructs in *Synechocystis*.** Using confocal microscopy the WT (*A*) was compared with strains expressing GFP fused to the signal sequences of the *E. coli* control TorA (*B*), *Synechocystis* FutA1 (*C*), *Synechocystis* FutA2 (*D*), and *Trichodesmium* Tery_3377 (*E*); the GFP control, *i.e.* without signal sequence, is shown in *F*. Imaging was performed under identical settings with excitation at 488 nm and is shown as GFP emission at 510–530 nm (*panels i*) and as autofluorescence (represented by phycocyanin) at 600–700 nm (*panels ii*); the merged images are shown in *panels iii*. Periplasmic localization of GFP is indicated by green fluorescent halos around the periphery of the cells in all strains (*B–E*) but not in WT (*A*) or the GFP control (*F*), with the latter showing cytosolic expression in absence of a signal peptide. The *scale bar* represents 1.5 μm.

The strains were visualized by confocal microscopy. Excitation of WT cells at 488 nm does not result in fluorescence emission in the GFP channel at 510–530 nm, but prominent autofluorescence from thylakoid membrane–localized photosynthetic pigments was observed when emission was monitored at 600–700 nm (Fig. 2*A*), as expected. Fusion of sfGFP to the TorA (Fig. 2*B*) or FutA2 (Fig. 2*D*) signal peptide results in bright fluorescent halos that surround the autofluorescence signal in merged images, indicating translocation of GFP across the cytoplasmic membrane to the periplasm. It is perhaps surprising that the FutA1 signal peptide also appears to localize some GFP to the periplasm or cytoplasmic membranes, albeit to a lesser extent than with the TorA or FutA2 fusions (Fig. 2*C*). Previous studies have suggested that FutA1 is mainly associated with thylakoid membranes, although it has been detected in the plasma membrane (26).

Fusion of the Tery_3377 signal peptide to GFP results in distinct halos (Fig. 2*E*), although this is not as clear as for the TorA and FutA2 signal peptides (Fig. 2, *B* and *D*) and also showed similarity to the FutA1 fusion (Fig. 2*C*). We changed the twin arginine residues in the Tery_3377 signal peptide to lysines, which prevented export of GFP and showed that the WT Tery_3377 signal peptide is recognized and exported by the twin-arginine translocase (TAT) system (Fig. S2). Immunoblots with an anti-GFP antibody identified a protein ∼3 kDa larger than sfGFP in the stain with the mutated signal peptide, consistent with the presence of the uncleaved signal peptide (Fig. S3*A*). In the strain producing just sfGFP without a signal peptide, clear fluorescence signal mapping to the cytoplasm was observed (Fig. 2*F*), as expected and as reported previously for yellow fluorescent protein (30).

These results indicate that the Tery_3377 signal peptide can translocate GFP to the periplasm using the TAT system. Both of the native *Synechocystis* FutA signal peptides also result in some degree of cytoplasmic membrane or periplasmic localization of GFP. Based on previous work FutA1 is considered to be predominantly intracellular; hence this result is unexpected. It is likely that the mature protein sequences also play a role in cellular localization, but attempts to investigate this in strains in which GFP was fused to the full proteins rather than just the signal peptides were unsuccessful because the strains were either genetically unstable or GFP was cleaved from the protein (data not shown).

### Tery_3377 is involved in Fe^3+^ transport across the cell membrane

**Figure 3.**
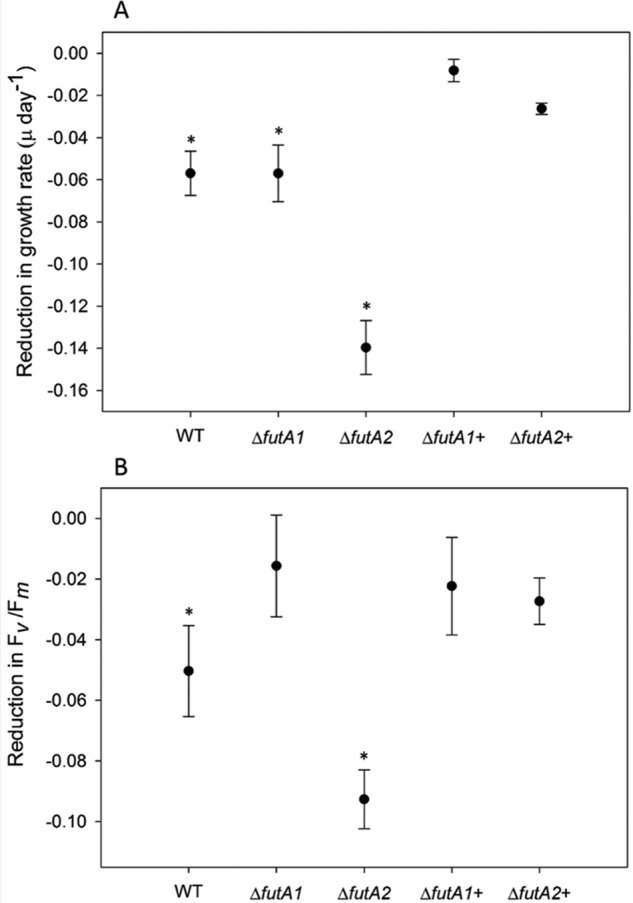
**Reduction in growth and photosynthetic efficiency of strains grown in the presence of ferrozine.** *A* and *B*, the decrease in growth rates measured as μ (day^−1^) (*A*) and photosynthetic efficiency measured as *F*_v_/*F*_m_ (*B*) during exponential growth of *Synechocystis* in YBG11 medium when grown with and without the Fe^2+^-binding ligand FZ. The data are presented for WT, Δ*futA1*, and Δ*futA2* mutants and the same strains expressing the *Trichodesmium tery_3377* gene (Δ*futA1*+ and Δ*futA2*+). *Error bars* show the standard deviation from the mean of three biological replicates. Statistical significance (*) was assessed using the Student's *t* test (*P* < 0.05).

The signal peptide fusions indicated that Tery_3377 is at least partly localized in the periplasm, presumably functioning as the periplasmic binding protein component of a ferric uptake ABC transporter. To gain further insight into the functional role of Tery_3377, the gene was codon-optimized for expression in *Synechocystis* and integrated at the *psbAII* locus in Δ*futA1* and Δ*futA2 Synechocystis* mutants. Production of Tery_3377 was confirmed by immunoblotting with the anti-IdiA antibody (raised against *Synechococcus elongatus* PCC 6302 IdiA) but was not detected using an antibody against *Synechocystis* FutA2 (Fig. 3*B*).

The high affinity iron ligand ferrozine (FZ) scavenges Fe^2+^ and cannot penetrate the cell membrane and thus is hypothesized to cause an iron-stress phenotype through reduction of ferrous iron uptake through the FeoAB system (31–33). This effect would be expected to be more severe in strains in which ferric iron uptake is also reduced, such as the Δ*futA2* strain. Experiments were performed in biological triplicate, and differences between FZ-treated and nontreated cells for each strain were determined for growth and photosynthetic efficiency (Fig. 3). A reduction in photosynthetic efficiency (*F*_v_/*F*_m)_ is expected under iron stress because of the essential role of iron in the photosynthetic electron transport chain (4, 5).

In the presence of FZ, growth rates were significantly reduced by 13% in the WT (*P* < 0.01) and 15% in the Δ*futA1* strain (*P* < 0.05; Fig. 3*A*), whereas the Δ*futA2* mutant lacking the Fe^3+^ transporter showed the greatest response, as expected (32% reduction, *P* < 0.01). The reduction in growth rate by FZ in both mutants was not as apparent when Tery_3377 was introduced to complement the knockout strains Δ*futA1* and Δ*futA2*, suggesting that Tery_3377 enables the cells to acquire ferric iron when ferrous iron is bound by FZ. Growth in presence of FZ also caused a reduction in photosynthetic efficiency (*F*_v_/*F*_m_) that was largest for Δ*futA2* (34%). The negative effect on the Δ*futA2* phenotype was again alleviated in the *tery_3377* complementation strain (Fig. 3*B*). In contrast, the reduction in photosynthetic efficiency in the Δ*futA1* knockout is less severe than WT.

### Tery_3377 binds ferric (Fe^3+^) iron

**Figure 4.**
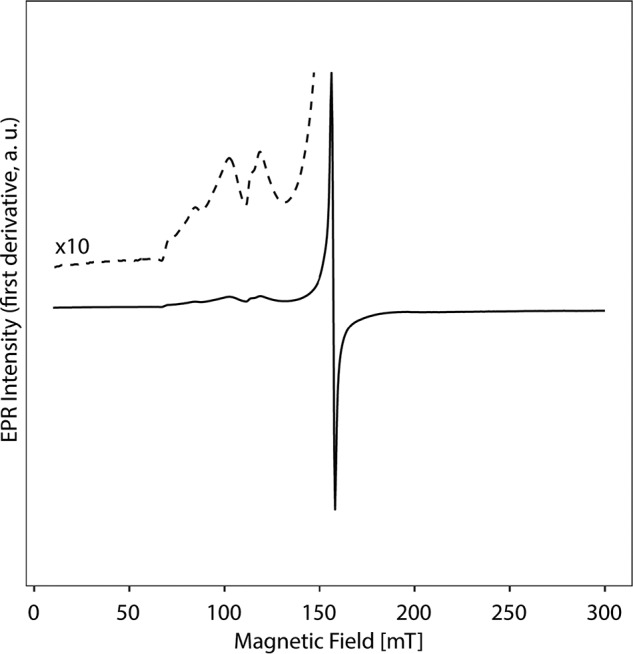
**EPR spectrum of reconstituted Tery_3377 probing the iron center.** Recombinant purified Tery_3377 from *T. erythraeum* ISM101 produced in *E. coli* was incubated with ammonium Fe(II) sulfate under aerobic conditions; the complex was repurified by size-exclusion chromatography to remove unbound iron. The EPR spectrum was collected from a sample with 0.55 mm protein concentration.

Recombinant Tery_3377 with a C-terminal His tag was overproduced in *E. coli* and purified to investigate its affinity and binding of iron species. After incubation with ammonium Fe(II) sulfate under aerobic conditions, the Tery_3377–iron complex was isolated by size-exclusion chromatography to remove unbound iron. The purified protein–iron complex was analyzed by EPR spectroscopy to determine the oxidation state of the bound iron ligand. EPR spectra revealed an oxidized Fe^3+^ state present in the Tery_3377 sample (Fig. 4). In addition to weaker signals between 800 and 1400 Gauss, a sharp and intense signal at ∼1500 Gauss (equal to a *g* value of 4.3) was clearly observed. This *g* value (4.3) is indicative of a high-spin Fe^3+^ ion bound to Tery_3377. The typical fingerprint weaker signals are related to higher order spin transitions of this ferric iron.

### Crystal structures of Tery_3377 reveal pentacoordination of Fe^3+^

To study Fe^3+^ binding by Tery_3377, the structure of the iron-bound protein was determined using X-ray crystallography (Table 1). The overall fold of Tery_3377 assumes a classic small molecule ABC transporter substrate-binding domain fold, with two domains hinged over a substrate-binding cleft. The hinge region is formed by two short β-strands and connects the protein domains via a double cross-over between the N (residues 34–133 and 266–349) and C termini (134–265). This type of structure places Tery_3377 among class IV iron-binding proteins (in keeping with Ref. 27) and allows for structural classification within cluster D of the substrate-binding domains (35, 36).

**Table 1:** **Crystallographic analysis: data collection and refinement statistics** The values in parentheses refer to the highest resolution shell.

	Tery_3377-Ala	Tery_3377-Water	Tery_3377-Cit
**PDB accession code**	6G7N	6G7P	6G7Q

**Data collection**			
Space group	P2_1_	P2_1_	P2_1_
Cell dimensions			
*a*, *b*, *c* (Å)	56.2, 83.2, 65.2	56.0, 82.9, 64.9	56.3, 102.0, 57.7
α, β, γ (°)	90, 95.20, 90	90, 95.20, 90	90, 112.85, 90
Resolution (Å)	29.26–1.1 (1.130–1.1)	29.11–1.5 (1.540–1.5)	28.64–1.2 (1.230–1.2)
*R*_merge_ (%)	0.04759 (0.3528)	0.04115 (0.2744)	0.07207 (0.8711)
*I*/σ(*I*)	11.83 (3.00)	16.22 (4.13)	11.63 (1.55)
*CC*½	0.998 (0.765)	0.999 (0.917)	0.999 (0.679)
Completeness (%)	99.42 (99.56)	95.48 (94.11)	98.30 (99.17)
Redundancy	3.2 (3.0)	3.5 (3.6)	5.8 (5.6)

**Refinement**			
Resolution (Å)	29.26–1.1 (1.129–1.1)	29.11–1.5 (1.539–1.5)	28.58–1.2 (1.231–1.2)
No. reflections	227,890 (16772)	85,685 (6274)	181,790 (13467)
*R*_work_ (%)	0.1337 (0.2358)	0.1476 (0.1790)	0.1355 (0.2630)
*R*_free_ (%)	0.1512 (0.2377)	0.1739 (0.2050)	0.1648 (0.2840)
Monomers/AU	2	2	2
No. of atoms	6236	5656	6198
Protein	5250	5106	5193
Ligand/ion	24	11	49
Water	962	539	956
*B*-factors	16.42	18.12	17.88
Protein	15.24	17.90	16.46
Ligand/ion	27.24	34.35	26.75
Water	29.35	24.75	31.17
R.m.s. deviations			
Bond length (Å)	0.012	0.018	0.019
Bond angle (°)	1.57	1.89	2.01

**Figure 5.**
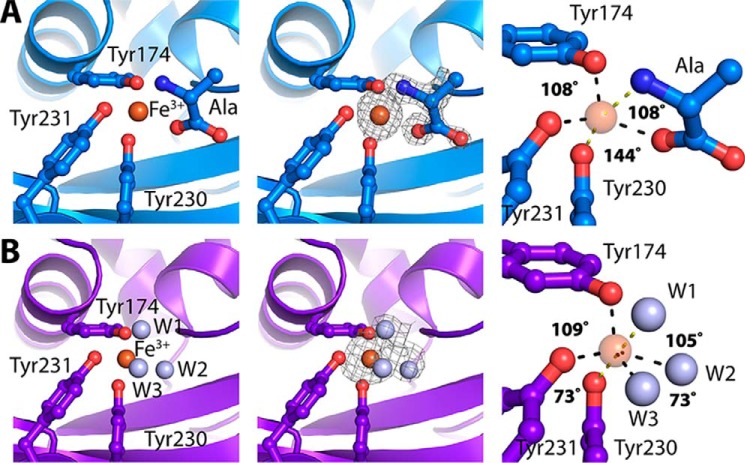
**Structure analysis of Tery_3377.** *A*, iron-binding site of Tery_3377-Ala with amino acid (*Ala*) ligand (*left panel*); the electron-density map shown (*middle panel*) is obtained after molecular replacement and subsequent refinement without ligands (*F*_O_ − *F*_C_, contoured at 3 σ); ligand coordination (*right panel*) shows iron in a trigonal bipyramidal geometry. *Red*, oxygen; *blue*, nitrogen; *brown sphere*, ferric ion; *yellow dashes*, H-bonds to axial coordination sites; *gray dashes*, H-bonds to equatorial coordination sites. *B*, iron-binding site of Tery_3377-water with water (*W*) ligands (*left panel*); the electron-density map shown (*middle panel*) is obtained after molecular replacement and subsequent refinement without ligands (*F*_O_ − *F*_C_, contoured at 3 σ); ligand coordination (*right panel*) shows iron in an octahedral geometry. *Red*, oxygen; *brown sphere*, ferric ion; *gray sphere*, water; *yellow dashes*, H-bonds to axial coordination sites; *gray dashes*, H-bonds to equatorial coordination sites.

Iron substrate–binding domains of different classes differ in iron ligand coordination, determined by conserved interacting amino-acid side chains of the substrate-binding domain, often involving tyrosine residues. In Tery_3377 three tyrosine residues, Tyr-144, Tyr-200, and Tyr-201, from the C-terminal domain contribute to iron binding. In the structure of Tery_3377-Ala (PDB entry 6G7N), determined at 1.1 Å resolution, a buffer molecule from the crystallization mixture occupies two coordination sites, giving rise to a pentacoordinated iron assigned as Fe^3+^ (Fig. 5*A*). The geometry observed is trigonal bipyramidal, with the buffer molecule alanine carboxyl group, Tyr-174 and Tyr-231, forming the trigonal plane with angles of 108°, 108°, and 144°. Tyr-230 and the amino group from the buffer alanine coordinate the axial iron positions.

Amino acid additives in the crystallization buffers lead to the most ordered and best diffracting crystals. However, Tery_3377 crystallized under a variety of conditions, including ones that lacked amino acid additives. We determined the structure of the protein crystallized with a monosaccharide additive mixture and found it was devoid of an iron-coordinating ligand in the binding site, with the iron instead coordinated by ordered water molecules (Fig. 5*B*). Comparison of the Tery_3377-water structure (PDB entry 6G7P) to the Tery_3377-Ala complex shows that water molecules W1 and W2 take the positions of the alanine amino and carboxylate groups of the alanine ligand, respectively (Fig. 5). Interestingly, in this complex an additional water molecule ligand (W3) is inserted between the coordinating oxygen atoms of W2 and Tyr-231. Compared with Tery_3377-Ala, where the observed angle between Tyr-23–1O and alanine-carboxyl oxygen was measured as 144°, this angle is split into two angles of roughly 73° measured between Tyr-231–O, the inserted water molecule W3, and W2. Like in Tery_3377-Ala, Tyr-230 and water W1 occupy the axial coordination sites in Tery_3377-water.

**Figure 6.**
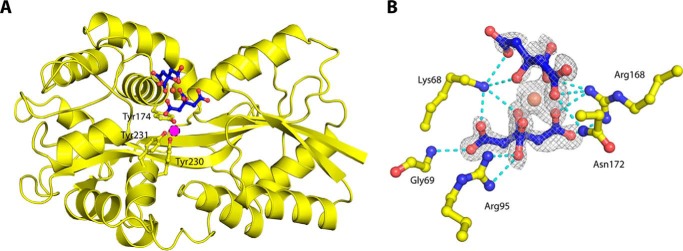
**Iron-binding site and coordination of citrate molecules.** *A*, the structure of Tery_3377-Cit. The fold is illustrated by the *yellow ribbon*, and the iron-binding site is shown by *sticks* representing the tyrosines shown in Fig. 5 (*yellow*) and two citrate molecules (*blue*). The iron coordinated by the two citrate molecules is shown as a *brown sphere*. The *magenta circle* indicates the position an iron ligand would occupy in iron-loaded Tery_3377. *B*, several protein amino acids, shown as *yellow sticks*, coordinate the citrate residues, shown in *blue*. The electron-density map is obtained after molecular replacement and subsequent refinement without ligands (*F*_O_ − *F*_C,_ contoured at 2 σ). *Red*, oxygen; *brown*, ferric ion; *gray mesh*, electron density; *blue dashes*, coordinating interactions.

To try and crystalline ligand-free Tery_3377 to examine structural changes upon iron binding, we citrate-treated Tery_3377 protein preparations prior to crystallization; citrate is known to chelate iron and was added to remove all iron from the protein preparation. The crystal structure (Tery_3377-Cit; PDB entry 6G7Q) traps the citrate molecule with bound iron in the substrate-binding cleft, where the iron is removed ∼7 Å from the binding site (Fig. 6). The iron-binding site (*pink sphere* in Fig. 6) was free of metal, and the significance of this finding in the context of possible siderophore acquisition is discussed below.

## Discussion

We have investigated the function and structure of *Trichodesmium* Tery_3377, an iron stress-induced protein (iron deficiency-induced A, IdiA) commonly used as an *in situ* marker of iron stress (15–17). Phylogenetic analysis (Fig. 1) groups Tery_3377 with the *Synechocystis* FutA1 protein, which has a suggested intracellular PSII protective function, rather than the FutA2 protein, a periplasmic protein with a role in iron transport. However, because of the high level of sequence identity of Tery_3377 to both FutA1 and FutA2 proteins and FutA1/2 proteins to one another, the function of these similar iron-binding proteins may be determined by their cellular localization. By fusion to sfGFP, Tery_3377 is shown to have a functional signal sequence for transport to the periplasm (Fig. 2) by the TAT system (Figs. S1 and S2). Consistent with this, Tery_3377 complements, the FZ induced the iron stress phenotype of the *Synechocystis* Δ*futA2* deletion strain (Fig. 3). These data support functional assignment of _3377 as a subunit of an iron-uptake ABC transporter.

Expression of Tery_3377 also complements the more subtle phenotype of the Δ*futA1 Synechocystis* mutant in the presence of FZ. This may indicate the possibility of an additional intracellular function in PSII protection or alternatively result from increased ferric iron uptake by Tery_3377. Because localization data using FutA1 signal peptide GFP fusions resulted in some periplasmic localization (Fig. 2), FutA1 may have a role in iron uptake in addition to its reported intracellular function. Indeed, despite their apparently distinct cellular locations and functions, a *futA1/futA2* double mutant in *Synechocystis* has more severe iron-deficient growth and ferric iron uptake phenotypes than either single mutant, suggesting some degree of redundancy (20). In agreement with this, the phenotype of the double mutant is similar to that of mutants of the single copy Fut ABC-transporter permease (*futB*) and ATPase subunit (*futC*) genes (20). Hence, the IdiA/FutA family of proteins may commonly display a dual function that requires a broader reassessment across cyanobacteria.

EPR spectroscopy and structural analysis confirms that Tery_3377 binds iron in the ferric oxidation state, Fe^3+^ (Fig. 4). Tery_3377 crystallizes under various conditions, often requiring small-molecule additives. The best diffracting crystals were obtained with an additive mixture of amino acids (Fig. 5*A*). An amino acid ligand, alanine, is identified in the iron-binding cleft; the amino acid is ordered and coordinates the iron substrate. In contrast, other crystallization conditions, which did not show ligand coordination by organic molecules, gave less well-ordered crystals that diffracted to lower resolution and generally refined to higher *R* values, indicating a lower quality of the resulting models (Table 1). We also determined the structure where organic ligands are absent from the iron-binding site, with three water molecules coordinating the iron, in place of the two coordinating atoms seen with the alanine ligand structure (Fig. 5*B*).

**Figure 7.**
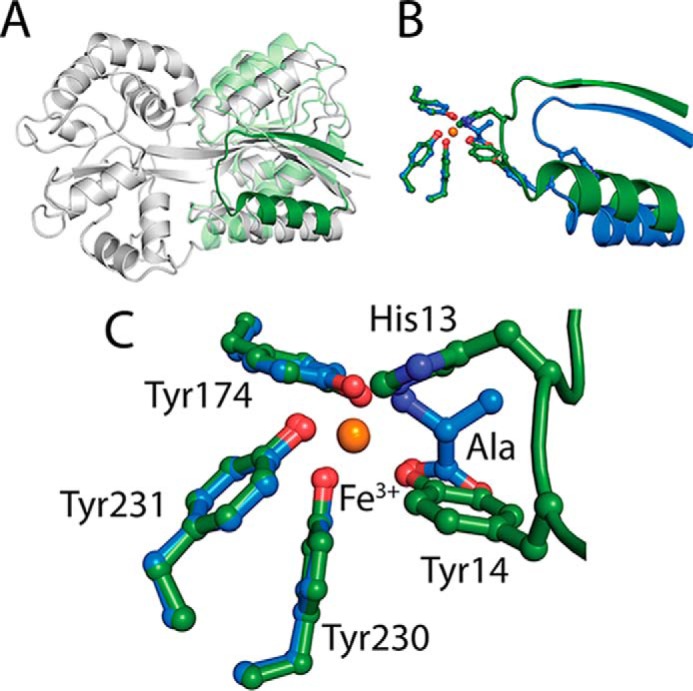
**Analysis of iron coordination in Tery_3377.** *A*, the open state of FutA2 from *Synechocystis* sp. PCC6803 is iron-free (*gray*). When superposed with the closed iron-bound form of FutA2 from *Synechocystis* sp. PCC6803 (Fe-FutA2), the C-terminal domains can be brought into perfect overlap, whereas the N-terminal domains reveal structural changes (*green* and *light green*) indicative of a closing of the substrate-binding cleft. Notably, a loop region contributing binding residues to the iron-binding sites is observed in a different position (*green*) to the open state. *B*, superposition of Tery_3377-Ala (*blue*) with Fe-FutA2 (*green*). The loop from the N-terminal domain brings iron coordinating tyrosine and histidine side chains into contact distance, allowing iron coordination. *C*, coordination site of superposed Tery_3377-Ala (*blue*) with iron–FutA2 (*green*). The equivalent amino acid residues to His-13 and Tyr-14, His-43, and Tyr-44 in Tery_3377 are not seen in contact with iron in either Tery_3377-Ala nor Tery_3377-water. However, the polar atoms from the alanine ligand in Tery_3377-Ala superpose well with the coordinating atoms of His-13 and Tyr-14. The three coordinating tyrosine residues originating from the C-terminal domains occupy the same position in Tery_3377-Ala and Fe-FutA2. Coordinating residues (shown as *sticks*) and ferric iron (*brown sphere*) are shown.

To determine the most likely coordination state of iron in Tery_3377, we superposed the Tery_3377-Ala structure with a classic closed conformation FutA2 structure (*Synechocystis* PCC6803 FutA2, PDB entry 2VP1) (Fig. 7*A*). The coordinating oxygen of the carboxylic acid moiety of the alanine ligand ideally superposes with a tyrosine side chain oxygen, whereas the amino nitrogen of the alanine ligand superimposes with a nitrogen from an interacting histidine ligand (Fig. 7, *B* and *C*). The two coordinating amino-acids in FutA2 are contributed by a loop that is brought into the vicinity of the iron-binding site by domain closure around the hinge that brings the two lobes of the substrate-binding domain closer together (37, 38) (Fig. 7*A*). Interestingly, this motif is conserved in Tery_3377, and such a domain closure, although not observed under any of our crystallization conditions, can be proposed. We conclude that the alanine ligand mimics this iron-binding mode, leading to a pentacoordinated state. This coordination state is corroborated by the low-temperature solution EPR spectroscopy, which revealed a strong signal at *g* = 4.3 and less intense higher-order transitions at higher *g* values, supporting generic class IV iron coordination by Tery_3377 in solution.

The observed binding of organic ligands together with iron may just be a fortuitous observation, but it might also have biological significance. It could be argued that the high concentrations of alanine present in crystallization mixtures out-competes interactions with the N-terminal iron-coordination residues His-43 and Tyr-44, thus leading to preferred crystallization of an open conformation. However, specific buffer molecules such as carbonate have previously been shown to coordinate iron in substrate-binding domains (39). Indeed, the binding site is large enough to fit a variety of amino acids in similar fashion to the shown alanine in Tery_3377-Ala. Hence, iron binding might concur with binding of different, larger molecules in the binding site. Nitrogen-containing molecules might enrich in the periplasm, creating a concentration gradient over the outer membrane. Additional support for the plasticity in the binding of Tery_3377 comes from the observation of bound citrate in the binding cleft, clearly indicating the potential for binding of organic compounds (Fig. 6). It is interesting to speculate that binding of organic ligands together with iron may represent a mechanism of siderophore recognition.

### Summary

This study provides structural and mechanistic insight into a key protein important in iron acquisition and the iron stress response in the globally significant marine diazotroph *Trichodesmium*. These organisms are central to N_2_ fixation, a highly significant process in the biogeochemical cycle, whose biogeography is determined by the availability of iron. Using heterologous expression in the model cyanobacteria *Synechocystis* PCC6803, we provide evidence that Tery_3377, a commonly used iron-limitation biomarker, is localized to the periplasm with a suggested FutA2 function as an iron-binding protein of an ABC transporter. However, Tery_3377 also complements deletion of FutA1, which could indicate that it also functions in protection of PSII, suggesting a potential dual function of Tery_3377 and more generally the IdlA/Fut family of proteins in cyanobacteria. Structural and spectroscopic characterization of recombinantly produced Tery_3377 confirms ferric iron binding under aerobic conditions. The structural data further show a remarkable plasticity of the substrate-binding cleft, allowing organic molecules and potentially siderophores to bind in addition to Fe^3+^.

## Experimental procedures

### Protein phylogeny analysis

Maximum likelihood phylogenetic analysis of protein sequences of Tery_3377 homologs from *Synechocystis* and selected bacteria was performed to identify possible functional clusters. Homologs of the *Trichodesmium* Tery_3377 and two *Synechocystis* proteins FutA1 and FutA2 were identified by searching the NR protein database with BlastP (40). The top 10 closest hits were retained for each query sequence and clustered at 90% identity using CD-HIT (41) to reduce redundancy. For each cluster, one representative was retained for inclusion in the analysis. Homologs from *Prochlorococcus marinus* MIT 9301, *S. elongatus* PCC 6301 and PCC 7942, and *Anabaena* sp. PCC 7120 were also included in the analysis as representatives of important primary producers, and sequences from *Haemophilus influenza* and *Neisseria meningitides* were chosen to represent pathogens.

The online platform Phylogeny.fr (42, 43) was utilized in “a la carte” mode to create an analysis pipeline. Sequences were first aligned with T-coffee (v11.00.8cbe486, (44), and the alignment was curated with G blocks (v0.91b) (45). Subsequently, a maximum likelihood phylogenetic tree was constructed using PhyML v3.1/3.0, (46), and an approximate likelihood ratio test was carried out to compute branch supports (approximate likelihood ratio test) (47). Finally, a graphical representation was produced with FigTree v1.4.3 (Institute of Evolutionary Biology, University of Edinburgh).

### Growth conditions

*Synechocystis* strains (Table S2) were routinely grown in BG-11 medium (48) supplemented with 5 mm glucose and buffered to pH 8.2 with 10 mm N-17 TES–KOH. Cultures were maintained at 30 °C with shaking at 150 rpm under constant illumination of ∼50 μm photons m^−2^ s^−1^. For growth on plates, 1.5% (w/v) agar and 0.3% (w/v) sodium thiosulphate were added. Antibiotics were included as appropriate (Table S2). FZ growth experiments were carried out in iron (0.6 mm FeCl_3_) YBG-11 media (recipe described in Ref. 49) at a FZ final concentration of 200 μm (FZ+) or no added FZ (FZ−). Growth was assessed under photoautotrophic conditions (no glucose), in triplicate cultures illuminated by 30 μm photons m^−2^ s^−1^ of red light to minimize abiotic photoreduction of iron in the presence of FZ (50). *E. coli* (Table S2) was grown shaking (250 rpm) at 37 °C in LB (Luria–Bertani) broth with 100 μg ml^−1^ ampicillin. The plates were set with 1.5% (w/v) agar.

### Photosynthetic physiology

Photosynthetic efficiency of *Synechocystis* strains during FZ growth experiments was measured as *F*_v_/*F*_m,_ an estimate of the apparent PSII photochemical quantum efficiency (51), using a FASTtracka^TM^ MkII fast repetition rate fluorometer integrated with a FastAct^TM^ Laboratory system (Chelsea Technologies Group Ltd., Surrey, UK). The samples were dark adapted for 20 min prior to measurements. The presented results were calculated from the average of three technical replicates for each of three biological replicates.

### Generation of Synechocystis strains

In this study, 1) pET21a was used for protein production in *E. coli*; 2) pACYC184 was used as a template to amplify the chloramphenicol resistance cassette; 3) pZEO was used as a template to amplify the zeocin resistance cassette; 4) pPD–FLAG was used as delivery vector for the introduction of genes into the *Synechocystis* genome in place of psbAII; and 5) pED151 was used for isolation of sfGFP.

For the generation of signal peptide fusions to sfGFP, DNA sequence corresponding to the predicted (in the case of *futA1*, *futA2*, *Tery_3377*) or known (*E. coli torA*) (52) signal sequence and the first 4 amino acid residues of the mature protein (as identified by SignalP v4.1/v3.0 sensitivity and TatP v1.0 servers (53, 54) (Table S1 and Fig. S1) was amplified from the respective organisms genomic DNA (for details of all primers used in this study see Table S3). Approximately 400 bp immediately preceding the *psbAII* gene was joined to each signal sequence by overlap extension (OLE)–PCR such that the ATG start codon of the signal sequence replaced the ATG start codon of *psbAII*. Using the same technique the chloramphenicol acetyl transferase (*cat*) gene from pACYC184 (New England Biolabs) was inserted between the coding sequence for sfGFP (55) and ∼400 bp of sequence immediately downstream of *psbAII*. Finally, each *psbAII* upstream-signal sequence fragment was joined to the sfGFP-*cat-psbAII* downstream fragment, again using OLE–PCR. The psbAII locus is extensively utilized as a “neutral site” that will not result in polar effects following gene insertion and is commonly used for expression of recombinant genes (56).

To generate a variant of the Tery_3377 signal peptide in which the twin arginine residues were replaced by lysines (3377ss-KK-GFP), alternative primers were designed to anneal during OLE—PCR, and both contained the first 18 bp of the Tery_3377 signal sequence with changed nucleotide sequence between positions 13 and 18 (AAA AAA in the place of AGA CGA), such that the translated sequence codes for two lysine residues in the place of the two arginine residues of the TAT system.

*Synechocystis* was transformed as described in Ref. 57 with selection on chloramphenicol (8.5 μg ml^−1^). Genome copies were segregated by repeated streaking on BG11 plates with increasing antibiotic concentration up to 34 μg ml^−1^, and homozygous transformants were verified by PCR and sequencing (Eurofins MWG Operon, Ebersberg, Germany).

For the generation of slr1295 (Δ*futA1*) and slr0513 (Δ*futA2*) *Synechocystis* deletion mutants, the antibiotic resistance cassette for zeocin (ZeoR) from pZEO (58) or the *cat* gene were used. PCR products consisting of the sequence upstream of the *futA1*/*futA2* genes and the beginning of the ORF or the end of the ORF and downstream flanking DNA were amplified from *Synechocystis* genomic DNA. Using OLE–PCR, ZeoR or *cat* was inserted between the upstream and downstream PCR products to create a linear mutagenesis fragment. For *slr1295* (*futA1*) the entire gene apart from the first 22 bp was replaced with the zeo cassette. For *slr0513* (*futA2*) all but the first 66 bp and last 41 bp of the gene were replaced by the cat cassette. The deletion constructs were introduced into WT *Synechocystis* as described above, with selection and segregation on zeocin (2.5–20 μg ml^−1^) or chloramphenicol (8.5–34 μg ml^−1^). Full segregation of mutant strains was confirmed by PCR.

For expression of *tery_3377* in *Synechocystis*, the gene was codon-optimized (Integrated DNA Technologies Inc., Leuven, Belgium) and cloned into the NdeI/BglII sites of pPD-FLAG (56). Transformation of *Synechocystis* with the constructed plasmid integrates *tery_3377* into the *Synechocystis* genome such that expression is driven from the *psbAII* promoter and confers kanamycin resistance allowing selection of transformed cells using increasing concentrations of kanamycin (10–40 μg ml^−1^). Segregation of transformants at the *psbAII* locus was confirmed by PCR, and the sequence of the gene was verified by sequencing, as above.

### Fluorescence microscopy

*Synechocystis* cells producing sfGFP fusion proteins were visualized using a Leica SP5 LSCM confocal microscope (Leica Microsystems, Wetzlar, Germany) or a axioscope Z plus epifluorescence microscope (Zeiss, Oberkochen, Germany). For confocal microscopy, samples were excited at 488 nm, and emission was measured at 510–530 nm for sfGFP fluorescence or 600–700 nm for autofluorescence, as described in Ref. 59. For visualization of sfGFP by epifluorescence microscopy, excitation was set at 400–490 nm (max 441 nm), and emission was measured at 460–570 nm (max 485 nm).

### EPR

Oxidized Tery_3377 (550 μm) was placed in EPR quartz tubes (Wilmad) and shock-frozen in liquid nitrogen. X-band continuous wave EPR spectra were recorded on a Bruker eleXsys E500 spectrometer using a standard rectangular Bruker EPR cavity (ER4102T) equipped with an Oxford helium flow cryostat (ESR900). The spectrometer operates at X-band (∼9.4 GHz) frequency and uses a 1-mT modulation amplitude (peak to peak) and 2-milliwatt microwave power. The spectra were recorded at cryogenic temperatures (5–6 K). A typical spectrum was recorded in ∼15–30 min.

### Protein production, crystallization, and structure determination

To produce C-terminally His_6_-tagged Tery_3377 in *E. coli*, the ORF (minus the putative sequence encoding the N-terminal signal peptide and stop codon) was amplified from the *Trichodesmium* genome. The resulting PCR product was digested and cloned into the NdeI/XhoI sites of pET21a(+) (Novagen). The sequence verified plasmid was introduced into *E. coli* BL21(DE3), and production of the recombinant protein was induced in cells grown to an *A*_600_ of 0.6 by addition of 0.4 mm isopropyl-β-d-thiogalactopyranoside, followed by growth for a further 4 h. The cells were harvested by centrifugation (6000 × *g*, 20 min, 4 °C), resuspended in binding buffer (50 mm Tris, 200 mm NaCl, 5% glycerol, 20 mm imodazole), and broken (sonication for 10 s on, 30 s off; 2 min process time), and the cell-free extract was isolated as the supernatant following centrifugation (200,000 × *g*, 45 min, 4 °C). His-tagged Tery_3377 was purified by affinity chromatography (1-ml HisTrap HP columns; GE Healthcare) and size-exclusion chromatography (HiLoad 16/60 Superdex 200; GE Healthcare).

Tery_3377 crystals were obtained in space group P2_1_ with two molecules in the asymmetric unit with slightly different packing between iron-bound and iron-free forms of Tery_3377. Hanging-drop vapor diffusion used conditions based on or optimized from the Morpheus screen (Molecular Dimensions). The reservoir composition leading to Tery_3377-Ala crystals was 0.1 m amino acids mix, 0.1 m buffer system 2, pH 7.5, 50% precipitant mix 1. Tery_3377-Ala data were collected at the Diamond Light Source Beamline I02 to a resolution of 1.1 Å. Tery_3377-water crystals were obtained with a reservoir composition of 0.12 m monosaccharides mix, 0.1 m buffer system 2, pH 7.5, 50% precipitant mix 1. Tery_3377-water data were collected at the Diamond Light Source Beamline I02 to a resolution of 1.5 Å. Tery_3377-Cit crystals were obtained with a reservoir composition of 0.1 m carboxylic acids mix, 0.1 m buffer system 2, pH 7.5, 50% precipitant mix 1. Tery_3377-Cit data were collected at European Synchrotron Radiation Facility Beamline ID30B to resolution of 1.2 Å.

The XDS package (60) was used to index, integrate, and scale diffraction data. The structures have been determined by molecular replacement with Phaser (61) using coordinates of apo-FutA2 from *Synechocystis* (PDB entry 2VOZ) (21) and were further refined with REFMAC5 (62). During refinement, hydrogens were added in their riding positions, and refinement was iterated with water building and model correction in Coot (63).

### Immunoblotting

*Synechocystis* cells were resuspended in 100 μl of 10 mm Tris-HCl, pH 7.3, and heated for 5 min at 100 °C followed by centrifugation at 10,000 rpm for 5 min at 4 °C. The samples were analyzed by SDS-PAGE on 4–12% NuPAGE acrylamide gradient gels (Life Technologies). Protein was blotted onto nitrocellulose membrane, which was blocked for 1 h in Tris-buffered saline with Tween 20 (TSB-T) and 2% ECL Advance blocking reagent (GE Healthcare). Antibody incubations (1 h) and membrane washes were performed in TBS-T. Anti-IdiA and anti-Slr0513 (FutA2) antisera were a kind gift from E. Pistorius (24, 34) and were used at concentrations of 1:5000 and 1:10000 respectively. Following application of ECL Advance detection reagent (GE Healthcare), chemiluminescence was visualized using a VersaDoc^TM^ CCD imager (Bio-Rad).

## Author contributions

D. P., M. M. M., A. H., A. J. B., T. S. B., and I. T. formal analysis; D. P., M. M. M., A. H., T. S. B., and I. T. validation; D. P., M. M. M., A.H., A. J. B., and I. T. investigation; D. P. and M. M. M. visualization; D. P., M. M. M., A. H., T. S. B., and I. T. writing-original draft; A. H., F. M., and I. T. conceptualization; A. H., C.M.M., T. S. B., and I. T. supervision; A. J. B. and F. M. methodology; F. M. and I. T. data curation.

## Supplementary Material

Supporting Information

## Footnotes

4 The abbreviations used are:

PSII: photosystem II
ABC: ATP-binding cassette
EPR: electron paramagnetic resonance
FZ: ferrozine
PDB: Protein Data Bank
contig: group of overlapping clones
sf: super-folder
TAT: twin-arginine translocase
TES: [tris(hydroxymethyl)methyl]-2-aminoethanesulfonic acid
OLE: overlap extension.
