# Supplementary material for: Structural and functional characterization of IdiA/FutA (Tery_3377), an iron-binding protein from the ocean diazotroph Trichodesmium erythraeum
Source: J Biol Chem. 2018 Sep 14;293(47):18099–109. doi: 10.1074/jbc.RA118.001929 (PMC6254336; doi:10.1074/jbc.RA118.001929)
Supplement: Supporting Information [file supp_293_47_18099__index.html]

Structural and functional characterisation of IdiA/FutA (Tery\_3377), an iron binding protein from the ocean diazotroph Trichodesmium erythraeum — Structure and Function of Trichodesmium Tery\_3377 — Structural and functional characterization of IdiA/FutA (Tery\_3377), an iron-binding protein from the ocean diazotroph Trichodesmium erythraeum — Structure and function of Trichodesmium Tery\_3377 — Supporting Information 

# Structural and functional characterization of IdiA/FutA (Tery\_3377), an iron-binding protein from the ocean diazotroph *Trichodesmium erythraeum*

## Supporting Information

- Supplemental data - Supplementary figures, Supplementary tables, Supplementary references.
