# Supplementary material for: Structural and functional characterization of IdiA/FutA (Tery_3377), an iron-binding protein from the ocean diazotroph Trichodesmium erythraeum
Source: J Biol Chem. 2018 Sep 14;293(47):18099–109. doi: 10.1074/jbc.RA118.001929 (PMC6254336; doi:10.1074/jbc.RA118.001929)
Supplement: Supporting Information [file supp_RA118.001929_135327_2_supp_195107_pf1vff.docx]

Supplemental data

**Structural and functional characterisation of IdiA/FutA (Tery_3377), and iron binding protein from the ocean diazotroph *Trichodesmium erythraeum*.**

Despo Polyviou^‡,1^, Moritz M. Machelett^§, ‡,1^, Andrew Hitchcock^‡,1,2^, Alison J. Baylay^‡^, Fraser

MacMillan^¶^, C. Mark Moore^‡^, Thomas S. Bibby^‡,3^ and Ivo Tews^§,4^

From the ^‡^Ocean and Earth Sciences, National Oceanography Centre Southampton, University of Southampton, Waterfront Campus, European Way, Southampton SO14 3ZH, UK, the ^§^Department of Biological Sciences, Faculty of Natural and Environmental Sciences Life Sciences, Building 85, University of Southampton, Highfield Campus, Southampton SO17 1BJ, UK, and the ^¶^School of Chemistry, University of East Anglia, Norwich Research Park, Norwich NR4 7TJ, UK

^1^The first authors should be regarded as Joint First Authors.

^2^Present address: Department of Molecular Biology and Biotechnology, University of Sheffield, Firth Court, Western Bank, Sheffield S10 2TN, UK.

Running title: Structure and Function of *Trichodesmium* Tery_3377

To whom correspondence should be addressed:

^3^Thomas S. Bibby, Tel.: +44 (0)23-8059-6446; E-mail: tsb@noc.soton.ac.uk.

^4^Ivo Tews, Tel.: +44 (0)23-8059-4415; E-mail: Ivo.Tews@soton.ac.uk.

**Table of contents:**

**Supplementary figures, S-2**

**Supplementary tables, S-6**

**Supplementary references, S-8**

**Keywords:** cyanobacteria, ABC transporter, crystal structure, microscopy, *Trichodesmium*, iron, IdiA, FutA, diazotroph, iron deficiency

**Supplementary figures**

**
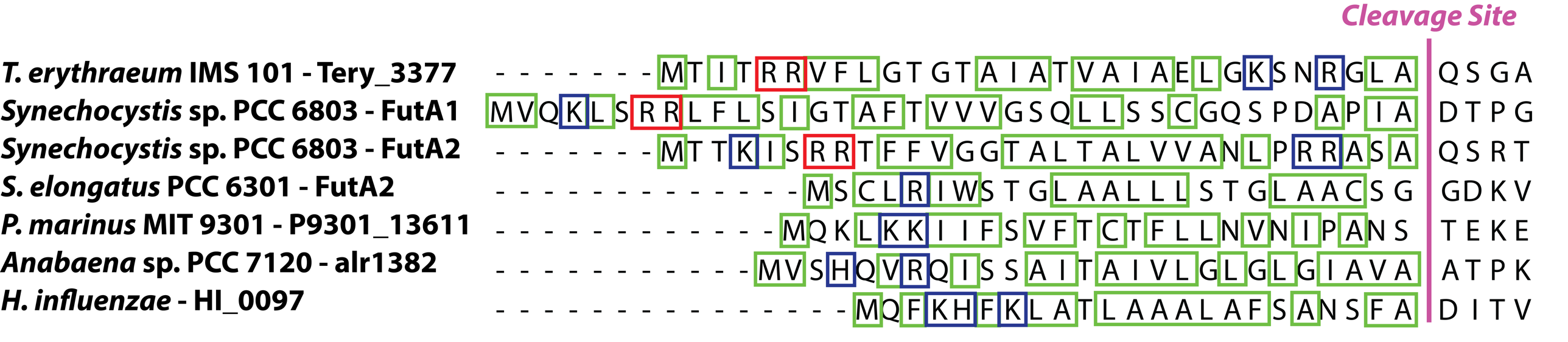
**

**Figure S1: Tery_3377 homologues of selected bacteria.**

Putative signal sequences as predicted from Signal P v4.1 and TatP. Twin arginine (RR) residues, hydrophibic residues (Kyte & Doolitle Hydrophobicity Scale) and positively charged residues of the signal sequence are identified. The cleavage site is indicated.

**Figure S2**: **The Tery_3377 N-terminal signal peptide targets GFP to the *Synechocystis* periplasm using the twin arginine translocase.**

*Synechocystis* WT **(A)** and TorAss-GFP **(B)**, Tery_3377ss-GFP **(C)** and Tery3377ss-KK-GFP **(D)** signal peptide fusion strains imaged by epifluorescence microscopy. Clear periplasmic haloes are present for the positive TorA signal peptide control and Tery_3377 signal peptide. Replacement of the Tery_3377 signal sequence twin arginine residues with lysines prevents periplasmic localisation, indicating that the protein is targeted to the TAT rather than the Sec system.

**A
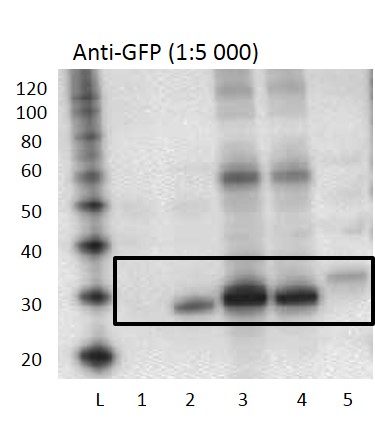
 B
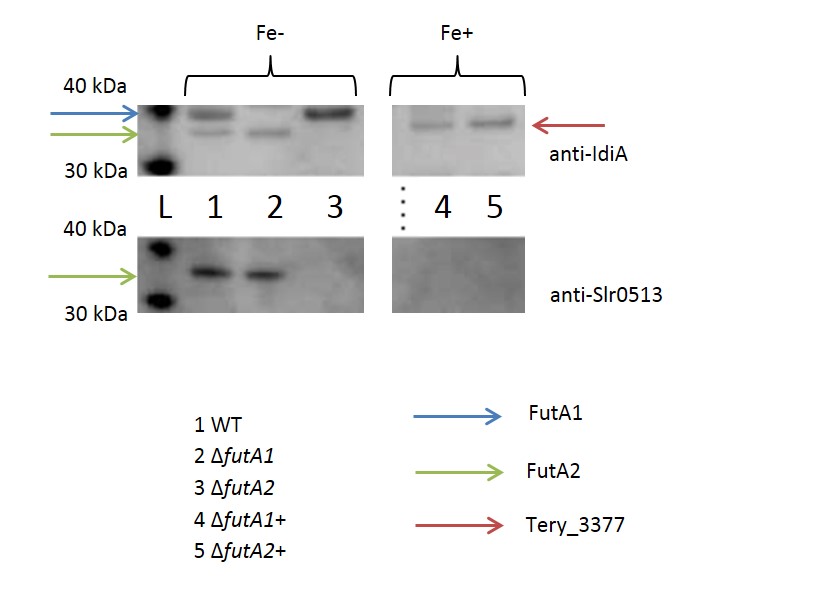
**

**Figure S3: Immunodetection of GFP, FutA1, FutA2 and Tery_3377 in *Synechocystis* strains used in this study. in *Synechocystis* strains**.

**(A)** Immunoblot analysis using anti-GFP on 3.5 μg protein samples (quantified using the Pierce™ BCA Protein Assay Kit, Thermo Scientific, DE, USA) extracted from WT *Synechocystis* (lane 1), and strains containing GFP only (lane 2), TorAss-GFP (lane 3), Tery_3377ss-GFP (lane 4) and Tery_3377ss-KK-GFP (lane 5). The box shows the signal corresponding to GFP, which is absent in the WT (lane 1). The TorA (lane 3) and Tery_3377 (lane 4) signal peptide GFP fusion strains show the same sized protein as GFP alone (lane 2). Conversely, in the strain with the mutated signal peptide fusion that is not recognized by the Tat system, a fainter band corresponding to GFP with the uncleaved signal peptide is detected (lane 5). Lane L = Ladder MagicMark™ XP (Thermo Fisher Scientific, Massachusetts, USA). **(B)** Anti-IdiA was raised against *Synechococcus elongates* PCC 6301 (Michel & Pistorius 1992) and cross-reacts with *Synechocystis* FutA1 and FutA2. Anti-Slr0513 was raised against *Synechocystis* 6803 FutA2 (Tölle et al., 2002); this antibody does not cross-react with FutA1. FutA1, FutA2 and Tery_3377 were identified in the different strains as expected. Protein extracts were acquired during Fe deplete growth for the WT and deletion mutants (lanes 1-3) and Fe-replete growth (to simplify identification of expressed Tery_3377 as FutA1 and FutA2 are not produced under such conditions) for the complemented strains **(4-5)**. Tery_3377 was only detectable using anti-IdiA, not anti-Slr0513. Gels were loaded with 3.5 μg protein / well for anti-IdiA blots and 1.75 μg protein / well for the anti-Slr0513 blot. Protein concentrations were assessed using the Pierce™ BCA Protein Assay Kit (Thermo Scientific, DE, USA) with bovine serum albumin (BSA) standards according to the manufacturer’s instructions.

**Supplementary tables**

**Table S1:** **Comparison of predicted signal peptides of Tery_3377 and FutA/IdiA homologues from other cyanobacteria.**

The presence of an N-terminal peptide in IdiA/FutA homologues was predicted using the Signal P v4.1 (v3.0 sensitivity) and TatP v1.0 servers. Where the servers disagreed on the length of the predicted signal peptide, the shorter prediction is shown in parenthesis. The localisation of the protein is stated if previously studied. The identity of each protein to Tery_3377 was identified through BlastP.

| Organism | Protein | SignalP prediction | TatP prediction | Length sig.seq. | Cellular location | Identity to  Tery_3377 (%) |
| --- | --- | --- | --- | --- | --- | --- |
| *Trichodesmium erythraeum IMS 101* | Tery_3377 | no | no | 31 (21) | unknown | N\A |
| *Synechocystis* sp. PCC 6803 | Slr1295 (FutA1) | yes | no | 37 | intracellular^1^ | 58 |
|  | Slr0513 (FutA2) | yes | yes | 31 | periplasm^1^ | 57 |
| *Synechococcus elongatus* PCC 6301 | syc0146_c (FutA2) | yes | no | 25 (22) | unknown | 57 |
|  | syc1920_d (IdiA) | no | no | 45 | intracellular^2^ | 54 |
| *Prochlorococcus marinus* MIT 9301 | P9301_  13611 | yes | no | 26 | unknown | 40 |
| *Anabaena* sp. PCC 7120 | Alr1382 | no | no | 27 | unknown | 30 |
| *Haemophilus influenza* | HI_0097 | yes | no | 23 | periplasm^3^ | 30 |

**^1^**Fulda *et al*., 2000; Tölle *et al*., 2002; Waldron *et al*., 2007.

**^2^**Michel *et al*., 1998.

**^3^**Adhikari *et al*., 1995; Kirby *et al*., 1997; Anderson *et al*., 2004; Khambati *et al*., 2010.

**Table S2.** Bacterial strains used in this study. Gene insertions/deletions in each strain are indicated. The antibiotic (kanamycin (kan), chloramphenicol (cm) and zeocin (zeo)) and concentration is shown in brackets.

|  | **Gene deletion (Ab^R^ μg/ml)** | **Gene insertion**  **(Ab^R^ μg/ml)** | **Source** |
| --- | --- | --- | --- |
| ***Synechocystis* sp. PCC6603**  WT-G |  |  | Cereda et al., 2014 |
| Δ*futA1* | *slr1295* (zeo20) |  | This study |
| Δf*utA2* | *slr0513* (cm34) |  | This study |
| Δ*futA1+* | *slr1295 (zeo20)* | *Tery_3377* (kan40) | This study |
| Δ*futA2+* | *slr0513* (cm34) | *Tery_3377* (kan40) | This study |
| FutA1ss-GFP |  | *slr1295ss-GFP* (cm34) | This study |
| FutA2ss-GFP |  | *slr0513ss-GFP* (cm34) | This study |
| 3377ss-GFP |  | *Tery_3377ss*-*GFP* (cm34) | This study |
| 3377ss-KK-GFP |  | *Tery_3377ss* (R5/6K)-*GFP* (cm34) | This study |
| ***Escherichia coli*** |  |  |  |
| XL1-Blue |  |  | Agilent Technologies, Stockport, UK |
| BL21(DE3) |  |  | New England Biolabs Ltd, Hitchin, UK |
| DH5-alpha |  |  | New England Biolabs Ltd, Hitchin, UK |

**Table S3.** Primer used in this study.

| Construct | Primer | Sequence |
| --- | --- | --- |
| futA1 deletion | P1  P2  P3  P4  P5  P6  zeo_F  zeo_R | CCAACCTCCAGTTCCACTGAC  ACATTAATTGCGTTGCGCTCACTGCGACGGGATAACTTTTGGACC  CAACTTAATCGCCTTGCAGCACATAGGTCCATTGGCAATGGTTG  GCCATTACGAAGGGGTGGACG  CGGCATTTTTCAGCCGGTGC  GGGCTGTGTCATTGCCCTGG  GCAGTGAGCGCAACGCAATTAATGT  ATGTGCTGCAAGGCGATTAAGTTG |
| futA2 deletion | P1  P2  P3  P4  P5  P6  cm_F  cm_R | AAGGCAGTGGTAAGGGCAAG  AAAGTTGGCCCAGGGCTTCCCGGTATACCACTAGGGCAGTGAGGG  CGATGAGTGGCAGGGCGGGGCGTAACCGATTCAGCTCGTTTAATG  GGGATTTCTTTGGCGGAGGT  AGCGTATCCAAAGTGAAGGGG  CGTGTCATTACCCTCCGGTT  TACCGGGAAGCCCTGGGCCAACTTT  TTACGCCCCGCCCTGCCACTCATCG |
| futA1ss-GFP | F  R | CAAATACATAAGGAATTATAACCAAATGGTCCAAAAGTTATCCCGTC  TGAACAGCTCTTCGCCTTTACGCATCCCTGGTGTGTCGGCGAT |
| futA2ss-GFP | F  R | CAAATACATAAGGAATTATAACCAAATGACAACTAAGATTTCCCGGCG  TGAACAGCTCTTCGCCTTTACGCATCGTGCGGGACTGGGCA |
| 3377ss-GFP | F  R | CAAATACATAAGGAATTATAACCAAATGACAATTACTAGACGAGTATTTC  TGAACAGCTCTTCGCCTTTACGCATTGCACCGCTTTGAGCTAAT |
| torAss-GFP | F  R | CAAATACATAAGGAATTATAACCAAATGAACAATAACGATCTCTTTCAGG  TGAACAGCTCTTCGCCTTTACGCATCGCCGCTTGCGCCGCA |
| psbAIIus | F  R | GGTATATGGATCATAATTGTATGCC  TTGGTTATAATTCCTTATGTATTTGTCGATG |
| psbAIIds | F  R | CGATGAGTGGCAGGGCGGGGCGTAAATTCCTTGGTGTAATGCCAACTG  CTGGTGGAAGCCCTGCGG |
| kan | F  R | TACCGGGAAGCCCTGGGCCAACTTT  TTACGCCCCGCCCTGCCACTCATCG |
| GFPss | F  R | ATGCGTAAAGGCGAAGAGCT  AAAGTTGGCCCAGGGCTTCCCGGTATCATTTGTACAGTTCATCCATACC |
| 3377ss-KK-GFP |  | ATGACAATTACTAAAAAAGTATTTCTAGGA |
| psbAII-us-R2 |  | ACTTTTTTAGTAATTGTCATTTGGTTATAATTCCTTATGTATTTG |
| 3377_OE | F  R | CATATGCAAAGCGGTGCAATTAATCTCTATT  tcagtggtggtggtggtggtgCTCGAGCTTCCAGCCTACACGATCCA |

**Supplementary references**

1. Michel, K.-P., Exss-Sonne, P., Scholten-Beck, G., Kahmann, U., Ruppel, H. G., & Pistorius, E. K. (1998). Immunocytochemical localization of IdiA, a protein expressed under iron or manganese limitation in the mesophilic cyanobacterium Synechococcus PCC 6301 and the thermophilic cyanobacterium Synechococcus elongatus. Planta, 205(1), 73–81.
2. Adhikari, P., Kirby, S. D., Nowalk, A. J., Veraldi, K. L., Schryvers, A. B., & Mietzner, T. A. (1995). Biochemical characterization of a Haemophilus influenzae periplasmic iron transport operon. Journal of Biological Chemistry, 270(42), 25142–25149.
3. Kirby, S. D., Gray‐Owen, S. D., & Schryvers, A. B. (1997). Characterization of a ferric‐binding protein mutant in Haemophilus influenzae. Molecular Microbiology, 25(5), 979–987.
4. Anderson, D. S., Adhikari, P., Nowalk, A. J., Chen, C. Y., & Mietzner, T. A. (2004). The hFbpABC transporter from Haemophilus influenzae functions as a binding-protein-dependent ABC transporter with high specificity and affinity for ferric iron. Journal of Bacteriology, 186(18), 6220–6229.
5. Khambati, H.K., Moraes, T.F., Singh, J., Shouldice, S.R., Yu, R.H. & Schryvers, A.B. (2010). The role of vicinal tyrosine residues in the function of Haemophilus influenzae ferric-binding protein A. The Biochemical Journal, 432(1), 57–64.
